# The impact of acute kidney injury on in-hospital mortality in acute ischemic stroke patients undergoing intravenous thrombolysis

**DOI:** 10.1371/journal.pone.0185589

**Published:** 2017-10-17

**Authors:** Florica Gadalean, Mihaela Simu, Florina Parv, Ruxandra Vorovenci, Raluca Tudor, Adalbert Schiller, Romulus Timar, Ligia Petrica, Silvia Velciov, Cristina Gluhovschi, Flaviu Bob, Adelina Mihaescu, Bogdan Timar, Goce Spasovski, Viviana Ivan

**Affiliations:** 1 Department of Nephrology, County Emergency Hospital Timisoara, Romania, ‘Victor Babes’ University of Medicine and Pharmacy, Timisoara, Romania; 2 Department of Neurology, County Emergency Hospital Timisoara, Romania, ‘Victor Babes’ University of Medicine and Pharmacy, Timisoara, Romania; 3 Department of Cardiology, County Emergency Hospital Timisoara, Romania, ‘Victor Babes’ University of Medicine and Pharmacy, Timisoara, Romania; 4 Department of Diabetes and Metabolic Diseases, County Emergency Hospital Timisoara, Romania, ‘Victor Babes’ University of Medicine and Pharmacy, Timisoara, Romania; 5 Department of Bioinformatics, County Emergency Hospital Timisoara, Romania, ‘Victor Babes’ University of Medicine and Pharmacy, Timisoara, Romania; 6 Department of Nephrology, Medical Faculty, University of Skopje, Skopje, Macedonia; Universidade Estadual Paulista Julio de Mesquita Filho, BRAZIL

## Abstract

**Introduction:**

Acute kidney injury (AKI) increases the risk of death in acute ischemic stroke (AIS) patients. Intravenous thrombolytic therapy (iv. rt-PA) seems to be the most effective treatment for AIS patients. The effects of AKI on iv. rt-PA treated AIS cases is less studied. Our paper addresses this issue.

**Methods:**

45 consecutive stroke patients treated with iv. rt-PA (median age = 64 years; 29 male) and 59 age and sex matched controls not eligible for iv. rt-PA have been enrolled in our study. Subjects were followed-up until hospital release or death (median follow up time = 12 days).

**Results:**

The prevalence of AKI did not differ between iv. rt-PA treated patients and controls (35.5% vs. 33.89%). In both groups, AKI was associated with increased in-hospital mortality: 50.0% vs. 3.4% p<0.0001 (in the rt-PA treated), and 45% vs. 30.7% (in controls). AKI iv. rt-PA treated patients had a significantly higher risk of in hospital mortality as compared to the no-AKI iv. rt-PA treated (HR = 15.2 (95%CI [1.87 to 124.24]; P = 0.011). In a Cox-multivariate model, the presence of AKI after iv. rt-PA remained a significant factor (HR = 8.354; p = 0.041) influencing the in-hospital mortality even after correction for other confounding factors. The independent predictors for AKI were: decreased eGFR baseline and elevated serum levels of uric acid at admission, (the model explained 60.2% of the AKI development).

**Conclusions:**

The risk of AKI was increased in AIS patients. Thrombolysis itself did not increase the risk of AKI. In the iv. rt-PA patients, as compared to non-AKI, those which developed AKI had a higher rate of in-hospital mortality. The baseline eGFR and the serum uric acid at admission were independent predictors for AKI development in the iv. rt-PA treated AIS patients.

## Introduction

Since it was approved in 1996 by the US Food and Drug Administration [1], the intravenous recombinant tissue plasminogen activator (iv. rt-PA) remains the most effective therapy for the acute ischemic stroke (AIS) despite its limited efficacy in patients with acute large cerebral artery occlusion [2]. The iv. rt-PA therapy can reverse neurological deficits, lower significantly the risk of long term mortality, and improve functional outcome, being at the same time a positive predictor of returning to full-time work after stroke [3, 4, 5].

Whether efficacy and neurological benefits of iv. rt-PA therapy are altered or not by preexisting renal dysfunction is still under debate. Pro [6,7,8] and con [9, 10] papers have been published lately.

In contrast, there are no data about the impact of the acute kidney injury (AKI) on the outcome of patients with AIS treated with iv. rt-PA, though there is an increased risk for AKI associated to acute cerebrovascular events [11, 12, 13, 14, 15]. We therefore performed a prospective observational study to report the incidence and predisposing factors for AKI in patients with AIS treated with iv. rt-PA and to evaluate the relation between AKI and in-hospital mortality in these patients.

## Material and method

### Ethics statement

The County Emergency Hospital Timisoara Ethical Committee (Board of Human Studies) approved the protocol (approval number 3/4th January 2013), and every patient provided written informed consent before enrolment. In those patients with impaired consciousness or aphasia, the written informed consent was obtained from a first–degree relative.

### Patients

The study was conducted and reported in accordance with the Strengthening the Reporting of Observational Studies in Epidemiology (STROBE) recommendations [16].

Prior enrolment sample size estimation was performed using data from literature (variations, standard deviations and minimum detectable difference). Based on this calculation, a number of 40 individuals in each group provided a statistical power of 80% and a corresponding α of 0.05 in case of no missing data, withdrawals or lost to follow-up.

From a pool of 1877 AIS patients admitted to the Neurology department between January 2013 and January 2015, we enrolled 45 consecutive cases treated with iv. rt-PA (with alteplase) up to 4.5 hours after stroke onset [17], (median age = 64 years, 64.44% (29) males, median time from stroke onset to treatment time = 150 minutes). The barrier to more frequent and successful thrombolysis was the lack of timely admission within the therapeutic window.

A sex and age-matched control group, including 59 patients (median age = 64 years, males 29 (49.15%), median stroke onset to door time = 360 minutes) was recruited from the large pool of AIS patients, ineligible for thrombolysis therapy because of their later referral. The control group received standard care.

No patients had concomitant organ failure and neither received nephrotoxic agents, at the time of enrollment.

Data concerning demographics and comorbidities (arterial hypertension—AHT, diabetes mellitus—DM, atrial fibrillation, coronary artery disease—CAD, peripheral vascular disease—PVD, prior stroke, congestive heart failure—CHF, dyslipidemia, alcohol consumption and smoke) have been retrieved from the GP’s files. Initial laboratory results including coagulation tests, complete blood count, glucose, uric acid and renal function (creatinine levels) were recorded. Serum creatinine values were measured on admission and routinely after that (usually daily), during hospitalization. A non-contrast head computed tomography (CT) was obtained and interpreted by a radiologist to evaluate for intracerebral haemorrhage (ICH) that would represent an absolute contraindication to iv. rt-PA. Stroke was defined according to the World Health Organization criteria [18]. The stroke subtype was defined on the basis of the TOAST criteria established by the Trial of Org 10172 in Acute Stroke Treatment (TOAST) classification [19]. Stroke severity was evaluated using the National Institutes of Health Stroke Scale (NIHSS) score [20]. Patients received iv. rt-PA ranging from 0.6 to 0.9 mg/kg, and the standard dosage was defined as 0.9 mg/kg. A second brain image by CT or magnetic resonance was routinely performed 24 to 36 hours after iv. rt-PA. In the case of clinical deterioration, patients underwent additional scans.

### Estimation of renal function and definition of AKI

Baseline renal function was assessed using the CKD-EPI creatinine equation [21]. AKI was defined and graded according to the Kidney Disease Improving Global Outcomes (KDIGO) criteria [22]. Serum creatinine at admission was considered to be the baseline. No patient required dialysis.

### Outcome measurements

Clinical outcome was in-hospital mortality and all patients were followed until hospital discharge or death. No patient was lost to follow-up.

### Statistical analysis

Data were collected and analyzed using the SPSS v.17 software suite (SPSS Inc. Chicago, IL, USA) and are presented as mean ± standard deviations for continuous variables with Gaussian distribution, median (interquartile range) for continuous variables without Gaussian distribution, or percentages for categorical variables. The lower and upper limits of the 95% confidence intervals (CI), used to estimate the prevalence, were calculated according to Wilson’s procedure for variables with Poisson distribution. Moreover, the 95% CI for odds ratio (OR) was calculated according to the mid-p method for binomial distributions. Survival was analyzed with the Hazard Ratio (HR) method and presented using Kaplan-Meier diagrams.

To assess the significance of the differences between groups, the Student *t*-test (means, Gaussian populations), Mann-Whitney-U test (medians, non-Gaussian populations), Chi-square (proportions) and log-rank test (differences between survival curves and hazard ratio) were used. Continuous variable distributions were tested for normality using the Shapiro-Wilk test, and for equality of variances using Levene’s test. For evaluating the involvement of more confounding factors in a time-related risk, Cox proportional-hazards models were built. Logistic regression analyses was performed to assess which parameters at admission may be considered as predictors for the development of AKI.

A p value of <0.05 was considered as the threshold for statistical significance.

## Results

The baseline characteristics of the studied groups are presented in Table 1. Demographic variables and stroke subtypes were similar in both groups (Table 1).

**Table 1:** Studied groups baseline characteristics.

Parameter	Thrombolysed group (n = 45)	Non-thrombolysed group (n = 59)	P
Age (years)^a^	64 [16]	65 [19]	0.24
Male gender (n[%])^c^	29 (64.44%)	29 (49.15%)	0.16
History of (n[%])^c^			
Cigarette smoking	22 (48.88%)	20 (33.89%)	0.15
Alcohol consumption	19 (39.58%)	10 (16.94%)	0.007*
Coronary artery disease	29 (64.44%)	38 (64.4%)	0.98
Arterial hypertension	33 (73.33%)	40 (67.8%)	0.69
Atrial fibrillation	8 (17.77%)	12 (20.33%)	0.80
Congestive heart failure	4 (8.88%)	6 (10.16%)	0.82
Previous ischemic stroke	1 (2.22%)	10 (16.94%)	0.021*
Diabetes mellitus	9 (20%)	15 (25.42%)	0.64
Dyslipidemia	23 (51.11%)	21 (35.6%)	0.16
Clinical and laboratory data on admission			
rt-PA dose (mg)^b^	71.21 ± 12.55	-	-
NIHSS on admission^a^	15 [6.5]	16 [6]	0.18
ASPECTS on admission^b^	9.2 ± 1.08	9.37 ± 1.05	0.54
SBP (mmHg)^b^	144.46 ± 18.04	142.54± 22.95	0.46
DBP (mmHg)^b^	79.13 ± 9.46	82.62 ± 12.53	0.07
BMI (kg/m2)^b^	27.16 ± 4.12	28.4 ± 4.34	0.14
Blood glucose (mg/dl)^b^	117 [33]	123 [71]	0.15
Serum creatinine (mg/dl)^b^	0.93 ± 0.44	1.04 ± 0.66	0.57
eGFR (ml/min/1.73m2)^b^	82.75 ± 20.51	77.25 ± 25.58	0.22
Serum cholesterol (mg/dl)^b^	196.17±46.31	179.84± 49.17	0.06
Serum triglycerides (mg/dl)^a^	130 [67]	104 [100]	0.07
Uric acid (mg/dl)^b^	5.60 ± 1.57	5.54 ± 1.97	0.65
Haemoglobin (g/dl)^b^	14.05 ± 1.36	13.55 ± 2.05	0.138
Hematocrit (%)^b^	42.2 ± 4.19	40.8 ± 6.34	0.20
Platelet count(x10^3^/μL)^b^	211.66 ± 54.89	249.6± 106.67	0.10
Stroke subtype (n [%])^c^			
Lacunar	9 (20%)	12 (20.33%)	0.96
Atherosclerotic	12 (26.66%)	16 (27.11%)	0.95
Cardioembolic	8 (17.77%)	11 (18.64%)	0.80
Cryptogenic	16 (35.55%)	20 (33.89%)	0.86
Simptomatic intracranial haemorrhage (n [%])^c^	9 (20%)	-	-
AKI (n[%])^c^	16 (35.5%)	20 (33.9%)	0.86
Death in 30 day (n [%])^c^	9 (20%)	21 (35.6%)	0.125

* Differences are significant

^a^ Variables with non-Gaussian distribution. Results are presented as median and [interquartile range]

^b^ Variables with Gaussian distribution. Results are presented as mean ± standard deviation. P was calculated using unpaired t-student test

^c^ Results are presented as number (percentage) from the group’s total. P was calculated using Fisher’s exact test.

rt-PA—recombinant tissue plasminogen activator; NIHSS—National Institutes of Health Stroke Scale; SBP—systolic blood pressure; DBP—diastolic blood pressure; BMI—body mass index; eGFR—estimated glomerular filtration rate

The prevalence of AKI in patients treated with iv. rt-PA was 35.5% (16 cases), being similar to that observed in the control group– 33.89% (20 cases) (P = 0.86).

The development of AKI in patients treated with iv. rt-PA was associated with increased baseline creatinine (1.2 vs. 0.8 mg/dL; P = 0.028) and baseline uric acid (6.7 vs. 5.0 mg/dL, P<0.0001) respectively decreased baseline eGFR (69.6 vs. 90.0 mL/min; P = 0.007). The comparison of other parameters between the patients with AKI and without AKI after iv. rt-PA is presented in Table 2.

**Table 2:** Comparison of parameters at admission between patients who developed vs. those who had not developed AKI, after iv. rt-PA.

Parameter		AKI (n = 16)	No-AKI (n = 29)	P
Age (years) ^a^		68.12 ± 7.98	64. 75 ± 12.83	0.342
Male gender (n[%])^b^		12 (75%)	17 (58.62%)	0.341
Creatinine at admission (mg/dL) ^a^		1.19 ± 0.66	0.8 ± 0.1	0.028*
Admission eGFR (mL/min) ^a^		69.6 ± 25.04	90.0 ± 13.1	0.007*
Hemoglobin (g/dL)^a^		14.5 ± 1.64	13.8 ± 1.1	0.105
Hematocrit (%)^a^		43.3 ± 4.85	41.5 ±3.59	0.165
Total cholesterol (mg/dL) ^a^		198.4 ± 39.4	195.0 ± 50.3	0.816
Triglycerides (mg/dL)^a^		163 ± 104.54	139.4 ± 65.85	0.42
Uric acid (mg/dL)^a^		6.7 ± 1.7	5.0 ± 1.1	<0.001*
Blood glucose (mg/dL) ^a^		147.93 ± 60.55	113.51 ± 22.3	0.11
SBP (mmHg)^a^		148.87 ± 14.74	142.0 ± 19.4	0.228
DBP (mmHg) ^a^		80.25 ± 7.27	78.5 ± 10.5	0.562
BMI (kg/m^2^)^a^		28.53 ± 3.35	26.4 ± 4.4	0.097
Stroke onset to thrombolysis time (minutes)^a^		138.1 ± 44.0	152.3 ± 46.2	0.320
rt-PA dose (mg)^a^		73.5 ± 10.0	70.0 ± 13.8	0.374
INR^a^		0.98 ± 0.06	0.95 ± 0.10	0.274
Platelet count(x10^3^/μL)^a^		221.81 ± 60.80	206.06 ± 51.61	0.388
Smokers (n[%])^c^^b^		11 (68.8%)	11 (37.9%)	0.046*
Alcohol consumption (n[%])^b^		10 (62.5%)	9 (31.0%)	0.041*
Coronary arterial disease (n[%])^b^		10 (62.5%)	19 (65.5%)	0.840
Arterial hypertension (n[%])^b^		12 (75%)	21 (72.41%)	0.851
Atrial fibrillation (n[%])^b^		2 (12.5%)	6 (20.68%)	0.691
Congestive heart failure (n[%])^b^		2 (12.5%)	2 (6.9%)	0.608
Previous ischemic stroke (n[%])^b^		0 (0%)	1 (3.4%)	0.644
Type 2 diabetes (n[%])^b^		5 (31.2%)	4 (13.8%)	0.161
Dyslipidemia(n[%])^b^		8 (50%)	15 (51.72%)	0.911
Admission NIHSS (score)^a^		16.25 ± 5.23	14.24 ± 4.01	0.15
Admission ASPECTS (score)^a^		9 ± 1.09	9.31 ± 1.07	0.366
Subtype of stroke (%) ^b^	lacunar	3 (18.75%)	6(20.68%)	0.876
	atherosclerotic	5 (31.25%)	7 (24.13%)	0.869
	cardioembolic	2 (12.5%)	6 (20.68%)	0.779
	cryptogenic	6 (37.5%)	10 (34.48)	0.839
Simptomatic intracranial haemorrhage (n [%])^b^		6 (37.5%)	3 (10.34%)	0.05
In hospital mortality (n [%])^b^		8 (50%)	1 (3.44%)	<0.0001

* Differences are significant

^a^ Variables with Gaussian distribution. Results are presented as mean ± standard deviation. P was calculated using unpaired t-student test

^b^ Results are presented as number (percentage) from the group’s total. P was calculated using Fisher’s exact test.

rt-PA—recombinant tissue plasminogen activator; NIHSS—National Institutes of Health Stroke Scale; SBP—systolic blood pressure; DBP—diastolic blood pressure; BMI—body mass index; eGFR—estimated glomerular filtration rate

**Fig 1:**
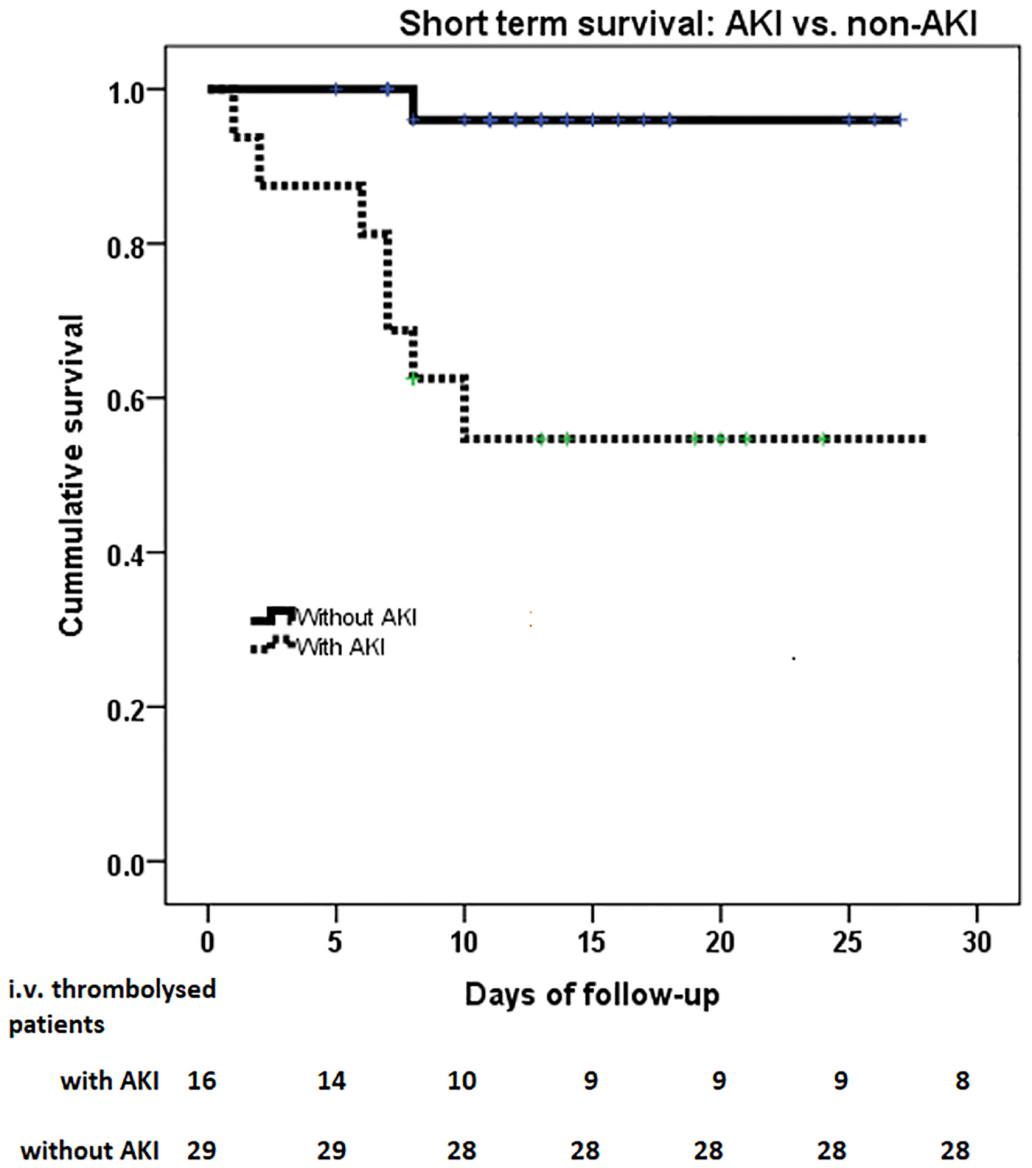
In-hospital survival in iv. rt-PA treated patients with AKI and those without AKI.

In the iv. rt-PA, treated group the development of AKI after thrombolysis was associated with an increased incidence of both overall in-hospital mortality (50.0% vs. 3.4%; P<0.001) and also in a time-to-event manner, observing a HR = 15.2 (95%CI [1.87 to 124.24]; P = 0.011). This finding highlights the worse short-term survival related to AKI in iv. rt-PA treated patients. (Fig 1)

The presence of AKI after iv. rt-PA remained a significant factor (HR = 8.354; p = 0.041) influencing the in-hospital mortality even after correction for other confounding factors in a Cox-multivariate model (in this model we included as covariates the presence of AKI, admission NISHH score, age andbaseline eGFR). The survival analysis and the time-series change of the number of subjects are presented in Fig 1.

To evaluate which parameters at admission may be considered predictors for the development of AKI in iv. rt-PA treated patients, we built a logistic regression model with predictors from the moment of admission (eGFR, uric acid, smoking status and alcohol consumption) and having as outcome the development of AKI. Our model explained 60.2% of the development of AKI (Nagelkerke R^2^ = 0. 602). According to our model, the eGFR value at admission and uric acid levels had a significant impact on this outcome, while the other parameters were not significantly associated (Table 3).

**Table 3:** Predictors for the development of AKI, accepted in the logistic regression model.

Predictor	B	OR [95% CI]	P
eGFR (per one ml/min/1.73m2 increase)	-0.69	0.93 [0.87 to 0.99]	0.044
Uric acid (per one mg/dL increase)	0.864	2.37 [1.15 to 4.91]	0.020
Smoking status	1.980	7.25 [0.58 to 89.8]	0.123
Alcohol consumption	0.877	2.40 [0.26 to 22.29]	0.440

eGFR—estimated glomerular filtration rate

## Discussion

To the best of our knowledge, this is the first study evaluating the association of AKI with in-hospital mortality in patients with AIS treated with iv. rt-PA. The thrombolysed patients who developed AKI were at a higher in-hospital mortality risk as compared with their non-AKI counterparts. The main finding of our work was that AKI is an independent prognostic marker for in-hospital mortality in patients with AIS treated with iv. rt-PA, even after adjusting for relevant covariates (age, baseline eGFR and stroke severity as assessed by NIHSS score).

Our study shows that the occurrence of AKI, defined by the KDIGO criteria, is a common finding in stroke thrombolysed and non-thrombolysed patients. More than one third of our patients developed AKI, the prevalence of AKI being similar in the patients treated with iv. rt-PA as compared to those non-thrombolysed (35.5% vs. 33.89%). The development of AKI was associated with low baseline eGFR and elevated levels of uric acid.

There are several published reports regarding the increased prevalence of AKI in patients with stroke (varying between 5.3% to 27.3%) [11, 12, 13, 14, 15, 23].

It seems that the development of AKI complicates the prognostic of stroke patients [11, 12, 13, 14, 15, 23]. AKI was also found to be an independent predictor for incident strokes. In a study performed by Wu et al., “recovery-AKI” patients turned out to have 1.3 times higher risk to develop future stroke attacks as compared to “no-AKI” individuals [24].

In our study, the prevalence of AKI was similar in both thrombolysed and non-thrombolysed patients which leaded us to the conclusion that thrombolysis itself is not associated with a higher risk of AKI development.

Data regarding the pathophysiology of renal changes secondary to neurological acute illness are sparse and several mechanistic explanations for the increased risk of AKI were presumed: hemodynamic variation in blood pressure, acute tubular injury from volume depletion, contrast induced nephropathy, and so on [13, 25].

In our study, AKI was associated with a significantly higher in hospital mortality rate After adjusting for available confounding variables, AKI remained an independent predictor of mortality in iv. rt-PA treated patients.

These results are similar with those reported by previous publications which have clearly demonstrated that AKI is an independent and strong predictor of both in-hospital and long-term mortality after stroke [11, 12, 13, 14, 15, 23]. Thus, in the patients who developed AKI after stroke, the mortality rate ranged between 42% [11] and 8.4% [13], being significantly higher as compared to AIS patients without AKI and the in-hospital mortality rates increase in parallel with the severity of AKI [11, 12, 14]. The observational design of our study does not allow conclusions concerning a direct causal relation between AKI and the excess mortality in AIS patients who had underwent iv. rt-PA.

The mechanism that leads to this heavy burden of mortality in patients with AKI is unclear and it is debatable whether AKI is a cause or a consequence of excess mortality [25 26]. Experimental and observational studies have shown that AKI is an acute systemic disease which induces distant organ injury in lung, brain, liver, intestine, heart and other vital organs [27]. Identified pathways of crosstalk between kidney and distant organ, include pro-inflammatory and pro-apoptotic cascades, oxidative stress, leucocyte activation, increased level of cytokines and chemokines, channel dysregulation, and regulation of cell-death in extra renal organs [27, 28]. It seems that reduced renal cytokine clearance and elevated cytokine production by the acutely injured kidney lead to a systemic inflammation status with negative effects on the distant organs [26]. These systemic phenomena associated with AKI predispose to multiple organ dysfunction and contribute to the higher mortality observed in patients with AKI [25]. In patients with AIS, AKI could aggravate the neurological lesions. Experimental published data revealed that AKI induced an increased vascular permeability with disruption of the blood-brain barrier, up-regulation of pro-inflammatory cytokines synthesis and granulocyte colony-stimulating factor and increase the microgliosis and neuronal pyknosis [27, 28].

On the other hand, it is possible that the higher mortality in AIS patients undergoing iv. rt-PA with AKI is associated with the complications of invasive procedures, which are more frequently required by AKI patients [13]. Further researches are required to determine if this is a causal relationship and if aggressive prevention of AKI development or treatment of the early signs of AKI, would result in decreased stroke mortality.

In the present study, we identified as independent predictors for AKI the following parameters: low eGFR level and elevated levels of uric acid. Several large epidemiological data have well established that reduced eGFR is a consistent risk factor for AKI [29, 30, 31]. In our study, we observed that higher levels of serum uric acid at admission represent an independent predictor for AKI. Recently, a meta-analysis analyzing data from 18 cohort studies with 75.200 patients have found that elevated serum uric acid levels were associated with an increased risk for AKI [32]. Furthermore, a large retrospective study of 14.535 hospitalized patients with cardiovascular, infectious disease, haematology/oncology, respiratory and gastrointestinal disorders, have demonstrated a linear relationship between serum uric acid level at admission and the development of AKI, with the highest risk in the admission serum uric acid >9.4 mg/dL patient group [33]. Also, for the AKI after cardiac surgery or contrast-induced AKI, elevated serum uric acid levels were frequently found to be associated with the risk of AKI developing [32, 34].

This study has several strengths. First, it is a prospective study and all consecutive patients who received iv. rt-PA for AIS at our hospital were included. Second, we included in the models the NIHSS score, which is a validated and widely used measure of stroke severity. Third, serum creatinine values were assessed during hospitalization and were used for these analyses. Furthermore, we used the standardized definition of AKI according to expert recommendations. However, our study has several limitations. First, this study is limited to a small number of cases. Although the number of inpatients with AIS during the study period was higher, only a few of them met the criteria required for the thrombolysis therapy. Second, evaluations of urine output were not available in all patients, which may contribute to an underestimation of the AKI events. Third, preadmission creatinine levels were not available in all cases. Fourth, we can’t analyse the effects of AKI stages on the outcome because there are few patients in the stage 2 and stage 3 of AKI.

## Conclusions

Our study shows that AKI is a common complication in patients with AIS. Thrombolysis itself was not associated with an increased risk for AKI. In the iv. rt-PA patients, as compared to non-AKI, those which developed AKI had a higher rate of in-hospital mortality. The baseline eGFR and the elevated serum levels of uric acid at admission were independent predictors for AKI development in the iv. rt-PA treated AIS patients.

These observations might have practical consequences in designing novel strategies to minimize the risk of AKI in stroke patients treated with iv. rt-PA in order to improve the outcomes of this medical treatment.

## Abbreviations

AHT: arterial hypertension
AKI: acute kidney injury
AIS: acute ischemic stroke
BMI: body mass index
CAD: coronary artery disease
CHF: congestive heart failure
CKD: chronic kidney disease
DM: diabetes mellitus
eGFR: estimated glomerular filtration
iv. rt-PA: Intravenous **t**hrombolytic therapy
ICH: intracerebral haemorrhage
KDIGO: Kidney Disease Improving Global Outcome
NIHSS: National Institutes of Health Stroke Scale
